# Behavioral Biometrics in VR: Changing Sensor Signal Modalities

**DOI:** 10.3390/s25185899

**Published:** 2025-09-20

**Authors:** Aleksander Sawicki, Khalid Saeed, Wojciech Walendziuk

**Affiliations:** 1 Faculty of Computer Science, University of Technology, 15-351 Bialystok, Poland; 2 Department of Computer Science and Electronics, Universidad de la Costa, Barranquilla 080002, Colombia; 3 Faculty of Electrical Engineering, University of Technology, 15-351 Bialystok, Poland; w.walendziuk@pb.edu.pl

**Keywords:** CNN, biometrics, deep learning, quaternion, VR, virtual reality

## Abstract

**Highlights:**

**What are the main findings?**

- Processing of orientation signals in the form of Euler angles, which is characterized by discontinuities, by CNNs is disadvantageous. Changing the modality of the trajectory and orientation time series into algebraically produced accelerometer and gyroscope signals has a positive influence on the effectiveness of the biometric system.
- The choice of neural network architecture has a significant impact on the identification metrics achieved. Sample processing by Multi-Input CNNs provides high classification accuracy. In the architecture mentioned above, acceleration and angular velocity signals are processed by separate branches with a set number of kernels. This makes it possible to determine the number of feature-extraction filters, which consequently enables equal consideration of selected modalities at the decision stage.

**What is the implication of the main finding?**

- It is possible to significantly increase the efficiency of person recognition by changing only the representation of the data. Sample modification is feasible for available data corpora and does not involve the collection of additional data (and therefore costs).
- The paper summarizes experiments involving several CNN architectures. On the basis of the compiled summary, the Multi-Input CNN is recommended for the processing of sets of varying modalities. This architecture performs feature extraction through filters separately for data processed within a branch.

**Abstract:**

The rapid evolution of virtual reality systems and the broader metaverse landscape has prompted growing research interest in biometric authentication methods for user verification. These solutions offer an additional layer of access control that surpasses traditional password-based approaches by leveraging unique physiological or behavioral traits. Current literature emphasizes analyzing controller position and orientation data, which presents challenges when using convolutional neural networks (CNNs) with non-continuous Euler angles. The novelty of the presented approach is that it addresses this limitation. We propose a modality transformation approach that generates acceleration and angular velocity signals from trajectory and orientation data. Specifically, our work employs algebraic techniques—including quaternion algebra—to model these dynamic signals. Both the original and transformed data were then used to train various CNN architectures, including Vanilla CNNs, attention-enhanced CNNs, and Multi-Input CNNs. The proposed modification yielded significant performance improvements across all datasets. Specifically, F1-score accuracy increased from 0.80 to 0.82 for the Comos subset, from 0.77 to 0.82 for the Quest subset, and notably from 0.83 to 0.92 for the Vive subset.

## 1. Introduction

Authorization methods in Virtual Reality (VR) systems are primarily based on traditional solutions such as PINs or passwords. In addition, two-factor authentication is increasingly being used, which increases the security level of systems. These approaches have three key drawbacks. First, sensitive data can be obtained by unauthorized persons, for example, as a result of shoulder surfing, i.e., peeking at the victim’s data as they enter it on the keyboard. Second, sensitive data may be deliberately shared with another person, which contradicts the basic principles of authorization [1,2]. Third, the need to enter passwords on the keyboard disrupts the feeling of immersion, which is a characteristic feature of this form of interaction [1]. It should also be noted that re-authorization (e.g., after a specific period of time) using a PIN negatively affects the usability of the VR system [3].

A review article [4] identifies head-mounted display (HMD) headsets as the most frequently used virtual reality devices, citing that their very concept (technological limitations aside) dates back to the 20th century. Examples of headsets listed include Lenovo Mirage, Oculus, Google Cardboard VR, HTC Vive, and FOVE, among others. The authors attempt to systematize existing solutions, grouping them into three categories. They distinguish solutions dedicated to hand, head, and full-body tracking. At the same time, significant attention is focused on behavioral biometrics related to eye movement analysis.

In [5] the authors present a comprehensive overview of the devices used for data acquisition. The publication addresses the issue of authorization in the field of Virtual Reality (VR), pointing to biometric analysis as one of the possible methods of securing access. The authors began the list with the commonly used Hand Held Controllers (HHCs), moving on to less conventional solutions. Among these, they listed additional gloves with haptic gloves, GSR (galvanic skin response) sensors, or electrodermal activity sensors, and HMD kits capable of measuring brain wave activity in the form of EEG (electroencephalography). It should be noted that in many cases, the data collected by HHC devices in the form of position and orientation is analyzed in a decision-making process based on artificial neural networks. The novelty of the approach presented in the paper is the artificial algebraic modality change for acceleration and position signals. This is intended to eliminate the heavily distorted orientation signals in the form of Euler angles.

Finally, it should be noted that the authors point to the development of whole-body tracking solutions, giving the example of the commercially available HaritoraX 1.1 system [6]. This device, in its basic version, consists of a set of five sensors, which work together with a set of manipulators and a VR helmet. Publication [7] presented an alternative approach to the data acquisition process. The described solution enabled the acquisition of information about the entire body posture, using resistive sensors. In the discussed work, the use of a suit was proposed across various application areas. These include, among others, gaming, virtual reality, rehabilitation, and educational applications such as dance learning.

Despite the inconvenience of wearing suits, they are usually more effective than vision systems, such as the Microsoft Kinect. This is particularly true in the area of limb orientation estimation [8]. In the field of behavioral biometrics in the VR area, data corpora have a wide variety of scenarios of activities performed. Among them (Table 1), we can distinguish: walking [9], throwing a virtual basketball [1], placing objects such as balls or cubes in containers [10], bowling and archery [2], pointing, grabbing, walking, and typing on a virtual keyboard [11], throwing a basketball [12] or the author’s own solution entitled “RubikBiom”, involving selecting a four-digit PIN on a virtual board [13]. Only some of the corpora allow more demanding cross-day validation [1,11,12]. In addition, it is worth noting that the effectiveness of the biometric system will be significantly affected by the number of labels in the area of a given collection, which typically ranges from 15 [10] to 41 [1].

The main idea behind the creation of behavioral biometric systems for VR is first of all to obtain an additional authorization mechanism, in the case where there is access to the VR system by an unauthorized person and they have acquired the password/PIN as a result of spying over the shoulder, intentionally, or under coercion from an authorized person. In such a situation, the biometric system can detect inconsistencies with the pattern in the way the person moves/executes movements.

It is worth noting that biometric systems can be validated in two scenarios. Single-day validation, carried out on training and test data from a single day, tends to be biased by priming and muscle memory [12]. In the case of data collected for two days, it is possible to draw conclusions based on data collected on one day and tested on another. This cross-day evaluation allows the biometric system to be verified under conditions close to real life.

The present work fills a gap in the field. Much of the work mentioned focuses on the aspect of the type of gesture made or the number of participants in the experiment. In contrast, few works focus on the development of methods for processing or changing the representation of available signals so as to increase the effectiveness of the biometric system for already collected data. In [3], the authors experimented with various types of signal normalization mechanisms, and in the present study, a step further was taken, and a modification was made by changing the representation of the signals.

## 2. Materials and Methods

For the present study, we used Miller’s open dataset [1,14,15,16]. The corpus contained recordings of basketball throwing motion in a virtual environment. As shown in paper [15] this type of motion carried an important biometric feature.

The corpus included motion recordings of 41 participants performing 10 repetitions of a virtual basketball throw, and such sessions were repeated over the course of two motion tracking sessions. In addition, participants used three different VR systems: Oculus Quest, HTC Vive Cosmos and HTC Vive, which resulted in three disconnected datasets. The dataset thus contained a total of about 2500 samples (41 × 10 × 2 × 3). The data was collected using two HHC manipulators and an HMD. The recordings of the projections made had a fixed length of three seconds. Due to different sampling rates, the recordings were 225, 135, and 135 frames long for each device.

**Figure 1:**
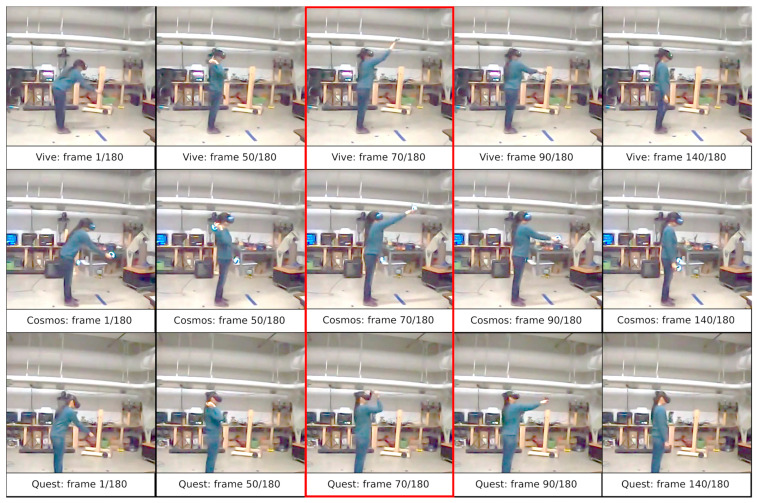
Summary of 5 keyframes for selected examples of footage collected by the Vive, Cosmos, and Quest devices. In the case of frame 70 (additional red border), there are large differences in the positioning of the right hand for the last of the VR systems. This demonstrates the varying dynamics of movement execution despite maintaining similar hand placements in frames 50 and 90.

Figure 1 presents a summary of selected frames from a video recording illustrating the making of a throw. The length of the recording is 3 s, which, at a sampling rate of 60 frames per second, results in a total of 180 frames. The analysis of the image sequence indicates that the termination of the motion usually occurs at frame 140, which corresponds to about 2.3 s. It should be noted, however, that due to the different speed of the executed motion, there is a situation where, for the 70th frame of the recording, depending on the iteration, there will be a different percentage completion of the sequence.

**Figure 2:**
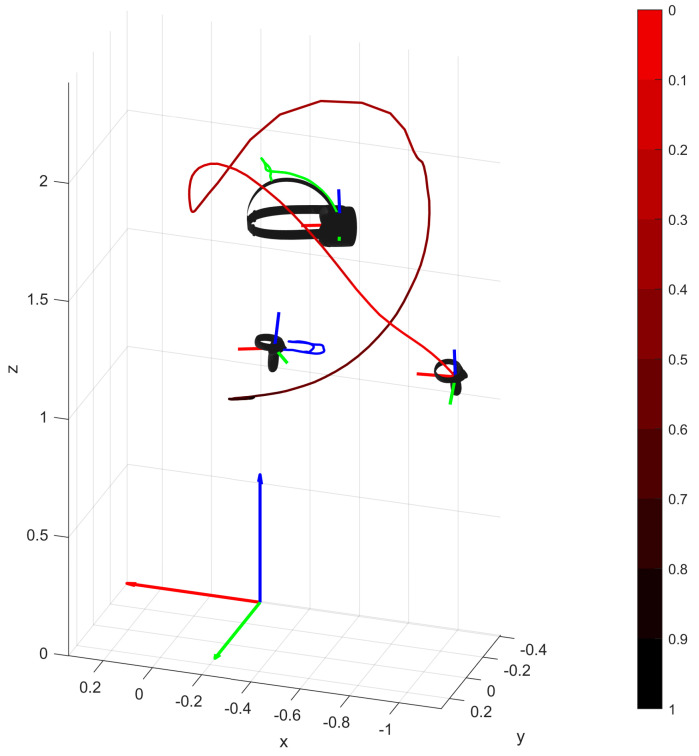
Visualization of the orientation and trajectory of the two HHCs and the HD display. In the case of the right controller, the trajectory of the object movement was visualized using shades of red. The light color marks the beginning of the movement, and the black color marks the end. In the final phase, the movement is performed more slowly than in previous steps of the conducted trial.

On the other hand, Figure 2 shows the motion trajectory and orientation of the HMD and the two HHC manipulators. The individual devices are shown as 3D objects. The graphics feature arrows representing the axes of the coordinate systems and broken lines reflecting the motion trajectories of the display and the two manipulators. The green color indicates the trajectory of movement of the display, and the blue color indicates the movement of the left manipulator.

In the case of the right-hand manipulator, the trajectory was visualized with an additional gradient. The initial position is marked in light red and the final position in black. Figure 2 shows that the typical movements of the head and left manipulator are small. The analysis of the color gradient of the right controller trajectory indicates that the movement in the initial phase was realized with high dynamics. In the final phase, however, the changes in position are small—the black color is practically invisible.

In addition, it should be noted that the movement of the right manipulator is performed along the *X* axis of the world system (which is the case for all of the participants). This implies a kind of “normalization” relative to the starting orientation. The invariability of this relative to the participants in the experiment is a significant simplification in the context of data preprocessing.

### 2.1. Methods

**Figure 3:**
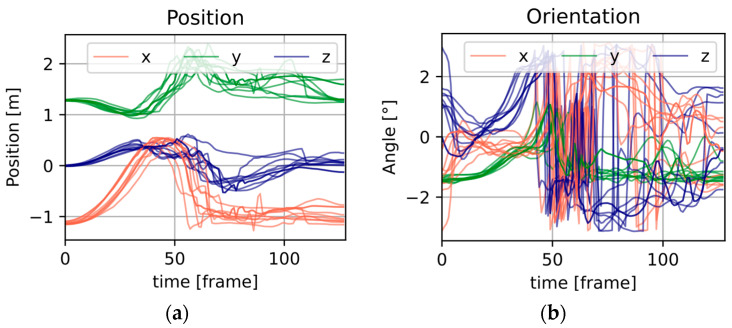
Measured values of (**a**) position and (**b**) orientation found in the original database.

The position and orientation signals of the controllers and the display were spaced in the data body. Direct use of the available signals is widely used due to implementation simplicity. In the case of building automatic detection systems, for example, using deep learning algorithms, the use of orientation signals described in terms of Euler angles is problematic due to the discontinuities that occur (Figure 3b). Therefore, it was decided to change the modality of the signals to IMU signals in order to minimize the artifacts.

In the literature, two main trends can be identified regarding the generation of IMU accelerometer and gyroscope signals using different measurement values. In the former, accelerometer and gyroscope signals are generated using differentiated ‘regression’ models. The applicability of this type of solution is limited by the requirement for prior training/mapping of the models. The learning set of such a solution must include samples of two modalities, e.g., IMU sensors and a depth camera. Among the representatives of the first group, it is worth mentioning articles [17,18]. In [17], the authors performed a bidirectional modality transformation of the samples between the IMU signals and the skeletal animation produced by the MS Kinect device. MIMO mapping was used to change the signal representation. In [18], on the other hand, regression models were adapted to transform the data from skeletal animation (the result of OpenPose software for video recordings) to IMU measurement data. The disadvantage of the above solutions is the requirement for two data modalities to be available at the learning stage. The Miller et al. data corpus does not include recordings of IMU signals, making it impossible to use this approach in further processing stages.

The second solution is to use an analytical approach. This makes it possible to determine the measurement values of the accelerometer and the gyroscope based on position and orientation time series. Among the representatives of the algebraic solution group, [19,20]. In [19], dedicated methods were implemented to generate acceleration (accelerometer) and angular velocity (gyroscope) data using position and orientation signals. A complementary solution developed by the MEMS Industry Group, within NXP, is presented in [20], in which case the intention of the developers was to create universal tools for testing data fusion algorithms. Additionally, in [21], the authors, based on [19], developed a solution for generating IMU data from recordings available on the YouTube platform. The described solution, designed for the area of Human Activity Recognition (HAR), was based on the extraction of motion trajectories and skeletal animation orientations from video recordings. Angular velocity or acceleration signals were then generated from these. The idea behind the presented solution was to provide a large amount of learning data using a rich set of source material. The deployment and mounting aspect of the sensors on the body was implemented to a small extent.

It could be assumed that open solutions such as [19,20] could be used as part of the ongoing work. However, their use encountered difficulties in setting up and running the working environment. Due to the limitations of the solutions [19,20], a partial modification of the [20] package in the Python programming language was carried out.

The IMUSim package [19] was abandoned in 2011 and was implemented in an unsupported version of the Python 2.X programming language. Running the package required bindings to use the Mayavi visualization. The easiest way to achieve this was through the Canopy software, which no longer supports pre-installed Mayavi binary packages.

**Figure 4:**
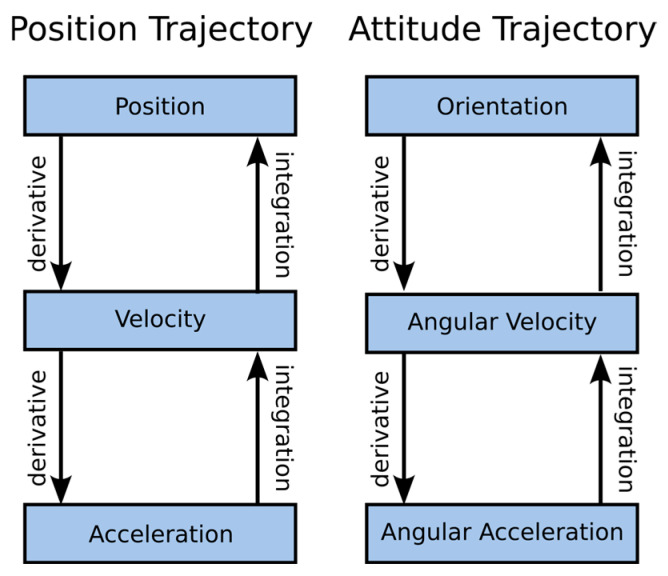
Block diagrams of T-sim solution trajectories [20].

On the other hand, the competing TSim was developed in the MATLAB programming language [20] (The code was published in accordance with MATLAB version 8.5). The main aspects that significantly hinder its use are that it cannot be simply ‘plugged’ into an existing data processing pipeline in Python programming environment ecosystem. In addition, the package makes it impossible to pass data for which uneven sampling has occurred. The software is based on the use of two types of trajectory, i.e., ‘Position Trajectory’ and ‘Attitude Trajectory’ [20], which are used to model acceleration and angular velocity signals (Figure 4). It can be seen in the figure that the linear acceleration information is calculated by processing the position signal and the angular velocity by processing the orientation trajectory.

In order to determine the artificially generated acceleration and angular velocity signals, it was necessary to know the position information *p*, the orientation *o*, and the sampling frequency. The position refers to a three-dimensional space according to:

*p* : *T* → ℝ^3^, *T* ∈ [0, *t_max_*] (1)

and the orientation is expressed by the quaternion relation:

*o* : *T* → *SO* (*3*), *T* ∈ [0, *t_ma_*_x_]. (2)

The velocity is determined using position differentiation, and the acceleration using double position differentiation:

*v*(*t*) = o*(*t*) ⊗ *p*’(*t*) ⊗ *o*(*t*) (3)

*a*(*t*) = o*(*t*) ⊗ *p*’’(*t*) ⊗ *o*(*t*) (4)

where ⊗ represents a Hamiltonian operator denoting a quaternion multiplication operation. Whereas * stands for a coupled quaternion.

Modeling of the gyro indication, i.e., angular velocity, is carried out according to the following formula

*ω*(*t*) = 2*M*(*o*) ⊗ *o’*(*t*) (5)

where the matrix *M*(*o*) is given by the formula:

(6) $$M\left(o\right)=\left[\begin{array}{cccc} -o_{x} & o_{w} & -o_{z} & o_{y}\\ -o_{y} & o_{z} & o_{w} & -o_{z}\\ -o_{z} & -o_{y} & -o_{x} & o_{w} \end{array}\right]$$

The differential of the quaternion, on the other hand, is determined according to the following formula:

(7) $$o’(t)\;=\;(o_{\left(t\;+\;1\right)}\;-\;o_{\left(t\right)})/∆T$$

**Figure 5:**
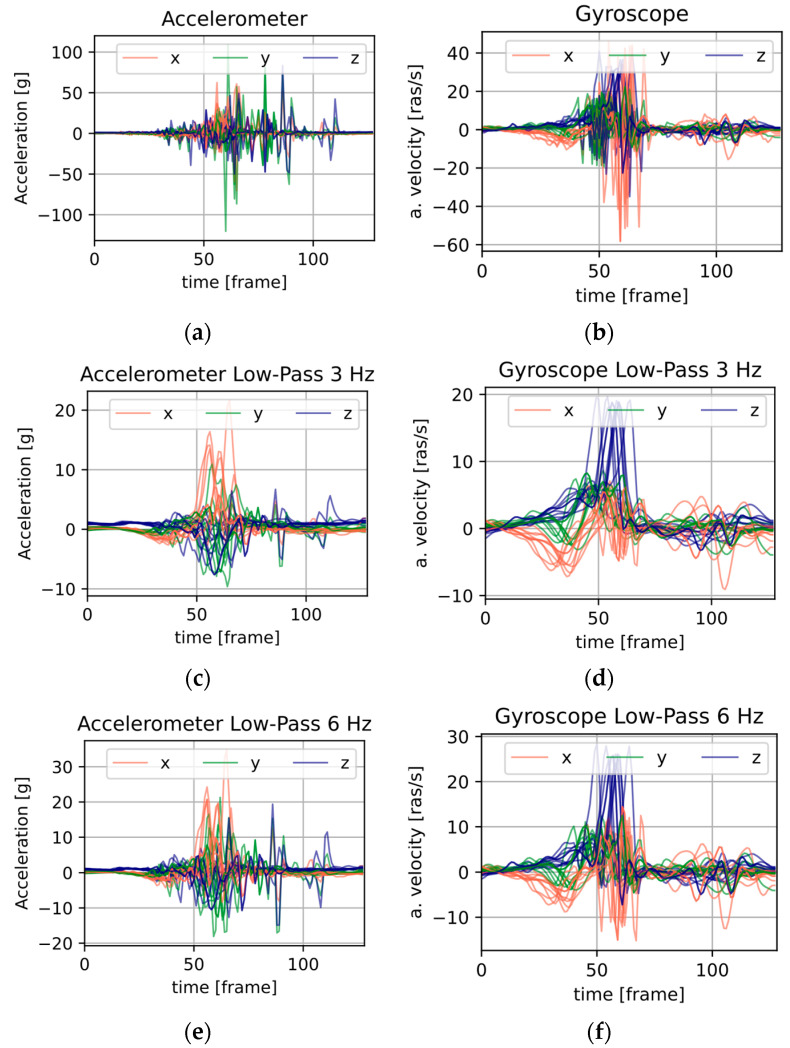
Values of acceleration (**a**) and angular velocity (**b**) signals; low-pass filtered with 3 Hz cut-off frequency (**c**) acceleration and (**d**) angular velocity; low-pass filtered with 6 Hz cut-off frequency (**e**) acceleration and (**f**) angular velocity.

Figure 5 shows a summary of the artificially produced signals. Each of the windows contains recordings from 10 motion repetitions, where a given modality has three channels (typically defined as *X*, *Y*, *Z*).

Figure 5a,b contain signals not subjected to frequency processing, and the former, as can be seen, has a much larger numerical range than the latter. Figure 5c,d show data subjected to low-pass frequency filtering with a cut-off frequency of 3 Hz, with Figure 5e,f showing 6 Hz. For these illustrations, the numerical ranges of both the acceleration and angular velocity data are similar.

### 2.2. Classifiers

As the largest position changes were recorded for the right controller, only information from this single device was used in further experiments. The limited and fixed duration of the recordings (the movement was recorded under laboratory conditions) allowed the use of CNN-type networks. Architectures of this type have enabled promising results in the field of motion recognition in VR [1,2,13] as well as related fields [20,21,22,23,24]. It is worth noting that deep learning models have been successfully applied to face recognition [25]. In the performed study, position and orientation time series, as well as algebraically generated accelerometer and gyroscope data, was used as input data.

The recording samples were represented in three-dimensional space, and the data was typically represented as arrays of 128 × 6. In the case of the Multi-Input CNN architecture, arrays of 100 × 3 were used as the input data of the two branches.

The cross-entropy typical of a classification problem was used as the cost function. Adam’s algorithm with a learning rate of 0.01 was used to optimize the network weights. The training process of the artificial neural network took 300 epochs. The architecture of the network is detailed in Table 1. Classification was carried out under 20 times simple validation. This was to minimize the impact of the randomness of the initial neural network weights on the results.

## 3. Results

The work carried out included a classification process using artificial neural networks. The experiments were carried out with data collected over two days, which means a more demanding cross-day validation. During the tests, the models were trained using samples collected over one day, and the validation of the developed methods was carried out using samples collected on day two. In order to minimize the randomness of the initial weights of the neural networks, the learning process was repeated 20 times. The F1-score metric was used as a measure of classification.

**Figure 6:**
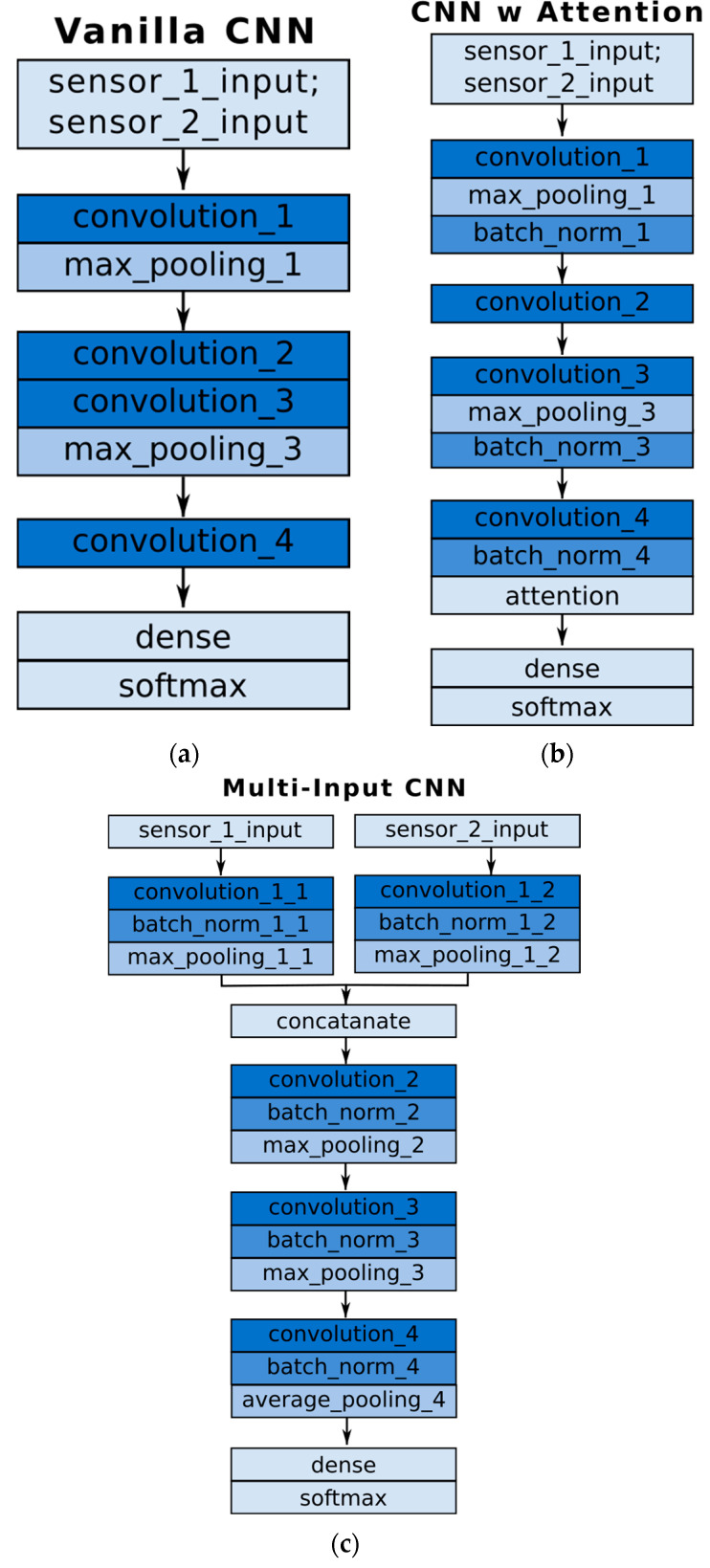
Vanilla CNN architecture (**a**) CNN with attentional mechanism (**b**) Multi-Input CNN (**c**).

Experiments were conducted for three variants of neural networks (i.e., Vanilla CNN, CNN with Attention, and Multi-Input CNN) for three subsets (acquisitions using Cosmos Quest Vive devices) and for four variants of input samples. Figure 6 shows the summary in heatplot form. The illustration shows three subgraphs representing the three tested neural network architectures. In each one, the vertical axis indicates the specific subset tested (depending on the acquisition device) and the horizontal axis—the tested modality. The first column on the left indicates the original data in the form of position and orientation, the second is the artificially generated modality of the accelerometer and gyroscope, and the next two columns differ in the filtering settings.

Figure 6 shows that the Vanilla CNN classifier achieves the lowest identification measure in the overall comparison. The CNN architecture with the attentional mechanism usually allows higher results, while the Multi-Input CNN classifier had the highest performance. Transforming modalities from the original data, such as position and Euler angles, to acceleration and angular velocity improves the identification accuracy of individuals. For the attention-based classifier, the highest results were obtained with a filtering cut-off frequency of 3 Hz, and for the Multi-Input CNN, with a setting of 6 Hz.

Applying the accelerometer and gyroscope signals in an unprocessed manner (ACC + GYRO columns) to the cases of Vanilla CNNs and CNNs with an attentional mechanism did not always yield the best results. Figure 5a,b show that the data is very noisy, and the accelerometer data can have twice the numerical range. This makes the gyro data in the inference process much less influential on the label prediction result. The use of frequency filtering smooths the accelerometer signals and reduces the disparity between the numerical ranges of the input data (Figure 5c–e). It should be noted that for the Multi-Input CNN architecture, this problem is minimized.

The results presented here show that the choice of decision model architecture has a very strong influence on the achieved result. In the conducted tests, the highest identification rates were achieved for the Multi-Input CNN model. This is the only model in which the use of the same number of kernels (feature extraction) from data from two separate branches was explicitly declared. This means that equal consideration was given to input data, such as accelerations and angular velocity.

**Figure 7:**
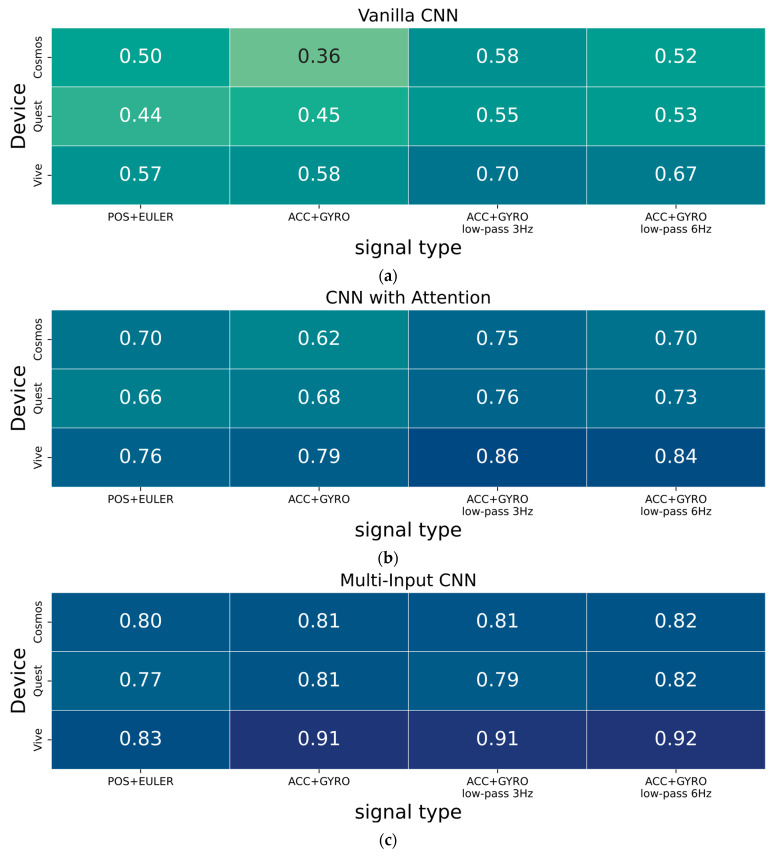
Heatplots of F1-score measure of Vanilla CNN classifiers (**a**) CNN with attentional mechanism (**b**) Multi-Input CNN (**c**).

Figure 7c shows that an efficiency of about 0.8 F1-score was achieved for the Cosmos and Quest device subsets, and 0.9 F1-score for the Vive. In the vast majority of scenarios analyzed, the lowest efficiency was observed for the Quest device subset. In our opinion, this is most likely due to multiple changes in the length of the recordings, which required alternating between decimation and interpolation of the data. The decrease in effectiveness is due to the loss of recording details, which reduced the number of biometric features.

The experimental results summarized in Figure 7 indicate that changing the modality of the signals positively affects the identification results. However, it is not possible to conclude from them whether the increase in performance is due to the elimination of only orientation (EULER) or also position (POS) signals. 

**Figure 8:**
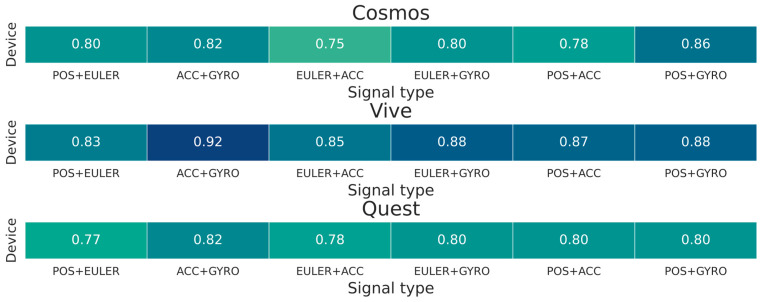
Heatplot diagrams for Multi-Input CNN classifier and additional modalities.

For a deeper analysis, additional experiments were conducted using the Multi-Input CNN classifier, which provided the highest classification measures in previous Experiments (Figure 8). Four additional trials for other modalities were added to the standard pairs of position and orientation (POS + EULER) and acceleration and angular velocity (ACC + GYRO) discussed. The performance was verified for the combined signals of orientation and acceleration (EULER + ACC), orientation and angular velocity (EULER + GYRO), position and acceleration (POS + ACC), and position and angular velocity (POS + GYRO). From the realized experiments, it can be seen that the use of the set of (EULER + ACC) and (EULER + GYRO) typically achieves low results. It should also be noted that the ACC + GYRO configuration achieved the highest measures for the two subsets recorded with the Vive and Quest devices.

Table 2, Table 3 and Table 4 provide a summary of the most commonly used biometrics metrics, such as AUC (Area Under the Curve), FAR (False Acceptance Rate), and FRR (False Rejection Rate). In the context of implementing the multi-class classification issue, metrics were developed for the One-vs-Rest (OvR) scenario. Analysis of the first table indicates that the Multi-Input classifier usually achieves the highest scores. Changing the modality leads to a slight increase in AUC—from 0.986 to 0.989 for the Cosmos corpus, from 0.991 to 0.998 for the Vive subset, and from 0.979 to 0.985 for the Quest subset. For the FAR metric, the lowest values were also obtained for the Multi-Input CNN classifier. Modifying the modality allowed the index to decrease slightly: from 0.0047 to 0.0046 for the Cosmos corpus, from 0.0038 to 0.0019 for the Vive subset, and from 0.0054 to 0.0045 for the Quest subset. These changes should be considered marginal. In contrast, for the FRR index, the lowest values were again observed for the Multi-Input CNN classifier. In this case, more significant differences were observed: from 0.1898 to 0.1838 for the Cosmos corpus, from 0.1515 to 0.0768 for the Vive subset, and from 0.2149 to 0.1795 for the Quest subset.

## 4. Conclusions

This paper presents the results of a study of person recognition based on the way right-hand movement is performed in the field of Virtual Reality. The experiments used an open data corpus of 41 participants containing motion recordings collected over two days. Data acquisition was conducted using Hand Held Controllers (HHCs) and head-mounted displays (HMDs). The data corpus contained three subsets, collected for variants of VR headsets (Cosmos, Quest, Vive).

Data acquisition for the collection was conducted in a way that mirrored the use scenario in the VR field. The motion required for repetition involved throwing a basketball, and participants used typical devices. In addition, the corpus allowed evaluation of the author’s experiments in cross-day validation (learning from day one data and validating on day two).

The research conducted investigated modality from the original position and orientation time series to algebraically created acceleration and angular velocity signals. Three typical CNN architectures were used in the evaluation. With the Multi-Input CNN variant, the highest person recognition rates were achieved. The proposed modality change increased performance from a 0.80 to 0.82 F1-score for the Comos set, from a 0.77 to 0.82 F1-score for the Quest system, and from a 0.83 to 0.92 for the Vive device (Figure 7). A major limitation of the present solution, but also of biometric systems in the behavioral field, is that the pattern of a feature changes over time. This forces continuous updating of reference samples and is a problem absent in the field of physiological biometrics. For example, in the gait domain, it is recommended that the dataset be updated every 9 months or so [26], a problem that also exists in the VR domain [16]. A consequence of this is that it is very often necessary to combine the two methods into hybrid solutions. Thus, as part of further work, it is planned to develop a solution in this field, i.e., using physiological features of the face and behavioral features, similarly to what was described in the article [27].

The novelty of the presented approach lies in the fact that it involves an algebraic change in signal modality to a form that provides higher classification rates. The proposed solution is not associated with an increase in cost, does not require, for example, the collection of additional samples, and provides an increase in the achieved results.

The experiments carried out showed that the standard position and orientation signals of the Hand Held Controller (HHC) can be effectively replaced by synthetic accelerometer and gyroscope data. The proposed modification has a favorable effect on the achieved performance indicators. The observed increase in effectiveness follows the replacement of “troublesome” (discontinuous) orientation signals in the form of Euler angles with another form of signals.

## Figures and Tables

**Table 1:** Comparison of existing behavioral biometric systems in VR.

Corpora Name	Acquisition Device	Number of People	Type of Movement	Description of Movement	Decision Model
GaitLock: Protect Virtual and Augmented Reality Headsets Using Gait [9]	Google Glass (HMD)	20	Gait	Indoor as well as outdoor gait. Data acquisition over two days, each time two sessions of motion tracking (direction of motion clockwise and counterclockwise) for a total of 4 sessions of gait	Hybrid decision-making model Dynamic-SRC (DTW + Sparse Representation Classifier)
VR-Biometric-Authentication [1]	Oculus Quest/HTC Vive/HTC Cosmos	41	Throwing a virtual basketball	Individuals perform a basketball toss in 10 attempts. There are two sessions, each held on a different day. Process repeated for 3 VR systems	Siamese network with a feature extractor in the form of CNN layers
BioMove: Biometric User Identification from Human Kinesiological Movements for Virtual Reality Systems [10]	HTC Vive (HMD, HHC); Glass (eye tracking device)	15	Placing objects-spheres/cubes in containers	Placing red balls into cylindrical containers and green/blue cubes into containers. Each participant completed 10 sessions per day over a two-day period.	k-Nearest Neighbors (kNN) and Support Vector Machine (SVM)
Understanding User Identification in Virtual Reality Through Behavioral Biometrics and the Effect of Body Normalization [2]	Oculus Quest	16	Implementation of two tasks: bowling and archery	Implementation of two task scenarios in a dedicated stage. This included task one for bowling and task two for target archery. Data collected within the two days	Deep Learning Classifier
Task-Driven Biometric Authentication of Users in Virtual Reality (VR) Environments [12]	HTC Vive	10	Basketball throw	Each action consists of four parts: picking the ball, placing the ball over the shoulder, throwing toward the target, and returning the controller/hand to the neutral position. During each session, 10 attempts/iterations were recorded	(nearest neighbor trajectory matching) similar to k-NN, *k* = 1
Knowledge-driven Biometric Authentication in Virtual Reality [13]	HTC Vive	23	RubikBiom’s proprietary solution. It involves selecting a 4-digit pin on a board in virtual reality	Choosing a number on a specially prepared three-color cube modeled in virtual reality (hence the reference to Rubik’s person). This cube had numbers, and the authors were asked to choose a sequence of 4 numbers with specific colors, e.g., 1 (green), 2 (white), 1 (red), 8 (white). Each participant entered 12 code sequences, assuming 4 repetitions	MLP FCN ResNet Encoder MCDCNN Time-CNN
Behavioural biometrics in VR: Identifying people from body motion and relations in virtual reality [11]	HTC Vive, eye tracking supplement by Pupil Labs	22	Perform four typical tasks in 3D environments: pointing, grasping, walking, and typing on a virtual keyboard	The task was carried out during two separate sessions over two days. With an interval of 3 days between acquisitions	Random Forest and Support Vector Machine (SVM)

**Table 2:** Mean AUC metrics for the analyzed subsets.

Area Under the Curve												
	Cosmos Subset				Vive Subset				Quest Subset			
CNN	0.949	0.896	0.954	0.944	0.965	0.952	0.974	0.970	0.926	0.898	0.939	0.929
CNN with Attention	0.979	0.972	0.986	0.982	0.986	0.978	0.986	0.985	0.970	0.962	0.975	0.971
M-I CNN	0.986	0.989	0.988	0.989	0.991	0.997	0.998	0.998	0.979	0.986	0.981	0.985
	POS + EULER	ACC + GYRO	ACC + GYRO L-P 3 Hz	ACC + GYRO L-P 6 Hz	POS + EULER	ACC + GYRO	ACC + GYRO L-P 3 Hz	ACC + GYRO L-P 6 Hz	POS + EULER	ACC + GYRO	ACC + GYRO L-P 3 Hz	ACC + GYRO L-P 6 Hz

**Table 3:** Mean FAR metrics for the analyzed subsets.

False Acceptance Rate												
	Cosmos Subset				Vive Subset				Quest Subset			
CNN	0.0111	0.0141	0.0090	0.0102	0.0093	0.0087	0.0065	0.0067	0.0125	0.0116	0.0096	0.0102
CNN with Attention	0.0071	0.0087	0.0060	0.0070	0.0052	0.0051	0.0034	0.0038	0.0083	0.0077	0.0057	0.0064
M-I CNN	0.0047	0.0045	0.0049	0.0046	0.0038	0.0021	0.0022	0.0019	0.0054	0.0043	0.0049	0.0045
	POS + EULER	ACC + GYRO	ACC + GYRO L-P 3 Hz	ACC + GYRO L-P 6 Hz	POS + EULER	ACC + GYRO	ACC + GYRO L-P 3 Hz	ACC + GYRO L-P 6 Hz	POS + EULER	ACC + GYRO	ACC + GYRO L-P 3 Hz	ACC + GYRO L-P 6 Hz

**Table 4:** Mean FRR metrics for the analyzed subsets.

False Rejection Rate												
	Cosmos Subset				Vive Subset				Quest Subset			
CNN	0.4432	0.5641	0.3617	0.4073	0.3709	0.3491	0.2611	0.2698	0.5010	0.4622	0.3844	0.4074
CNN with Attention	0.2823	0.3489	0.2387	0.2785	0.2078	0.2034	0.1341	0.1516	0.3330	0.3067	0.2279	0.2566
M-I CNN	0.1898	0.1817	0.1951	0.1838	0.1515	0.0843	0.0894	0.0768	0.2149	0.1739	0.1949	0.1795
	POS + EULER	ACC + GYRO	ACC + GYRO L-P 3 Hz	ACC + GYRO L-P 6 Hz	POS + EULER	ACC + GYRO	ACC + GYRO L-P 3 Hz	ACC + GYRO L-P 6 Hz	POS + EULER	ACC + GYRO	ACC + GYRO L-P 3 Hz	ACC + GYRO L-P 6 Hz

## Data Availability

The raw data supporting the conclusions of this article will be made available by the authors on request.

## References

[1] Miller R., Banerjee N.K., Banerjee S. Using Siamese Neural Networks to Perform Cross-System Behavioral Authentication in Virtual Reality. Proceedings of the 2021 IEEE Conference on Virtual Reality and 3D User Interfaces (VR).

[2] Liebers J., Abdelaziz M., Mecke L. Understanding User Identification in Virtual Reality through Behavioral Biometrics and the Effect of Body Normalization. Proceedings of the Conference on Human Factors in Computing Systems.

[3] Ajit A., Banerjee N.K., Banerjee S. Combining Pairwise Feature Matches from Device Trajectories for Biometric Authentication in Virtual Reality Environments. Proceedings of the 2019 IEEE International Conference on Artificial Intelligence and Virtual Reality (AIVR).

[4] Heruatmadja C.H., Meyliana, Hidayanto A.N., Prabowo H. Biometric as Secure Authentication for Virtual Reality Environment: A Systematic Literature Review. Proceedings of the 2023 International Conference for Advancement in Technology (ICONAT).

[5] Garrido G.M., Nair V., Song D. (2024). SoK: Data Privacy in Virtual Reality. Proc. Priv. Enhancing Technol..

[6] Shiftall Inc. HaritoraX 1.1/1.1B Online Manual (English). https://docs.google.com/document/d/14hY7mkejXRbMWGzry_tp7Xw7sEDwiQMw02C53XnVJe4/edit?tab=t.0#heading=h.21vh7twr76m1.

[7] Kim D., Kwon J., Han S., Park Y.L., Jo S. (2019). Deep Full-Body Motion Network for a Soft Wearable Motion Sensing Suit. IEEE/ASME Trans. Mechatron..

[8] Milosevic B., Leardini A., Farella E. (2020). Kinect and Wearable Inertial Sensors for Motor Rehabilitation Programs at Home: State of the Art and an Experimental Comparison. Biomed. Eng. Online.

[9] Shen Y., Wen H., Luo C., Xu W., Zhang T., Hu W., Rus D. (2019). GaitLock: Protect Virtual and Augmented Reality Headsets Using Gait. IEEE Trans. Depend. Sec. Comput..

[10] Olade I., Fleming C., Liang H.N. (2020). BioMove: Biometric User Identification from Human Kinesiological Movements for Virtual Reality Systems. Sensors.

[11] Pfeuffer K., Geiger M.J., Prange S., Mecke L., Buschek D., Alt F. Behavioural Biometrics in VR: Identifying People from Body Motion and Relations in Virtual Reality. Proceedings of the Conference on Human Factors in Computing Systems.

[12] Kupin A., Moeller B., Jiang Y., Banerjee N.K., Banerjee S. (2019). Task-Driven Biometric Authentication of Users in Virtual Reality (VR) Environments.

[13] Mathis F., Fawaz H.I., Khamis M. Knowledge-Driven Biometric Authentication in Virtual Reality. Proceedings of the Conference on Human Factors in Computing Systems.

[14] Wright State University TARS Open “VR-Biometric-Authentication” Repository. https://github.com/weibo053/VR-Biometric-Authentication/tree/4bbc555e6183327747cc29fd4463a71ab7894e09.

[15] Miller R., Banerjee N.K., Banerjee S. Combining Real-World Constraints on User Behavior with Deep Neural Networks for Virtual Reality (VR) Biometrics. Proceedings of the 2022 IEEE Conference on Virtual Reality and 3D User Interfaces (VR).

[16] Miller R., Banerjee N.K., Banerjee S. Temporal Effects in Motion Behavior for Virtual Reality (VR) Biometrics. Proceedings of the 2022 IEEE Conference on Virtual Reality and 3D User Interfaces (VR).

[17] Baños O., Calatroni A., Damas M., Pomares H., Rojas I., Sagha H., Millán J.D.R., Tröster G., Chavarriaga R., Roggen D. Kinect=IMU? Learning MIMO Signal Mappings to Automatically Translate Activity Recognition Systems across Sensor Modalities. Proceedings of the International Symposium on Wearable Computers (ISWC).

[18] Rey V.F., Hevesi P., Kovalenko O., Lukowicz P. Let There Be IMU Data: Generating Training Data for Wearable, Motion Sensor Based Activity Recognition from Monocular RGB Videos. Proceedings of the 2019 ACM International Joint Conference on Pervasive and Ubiquitous Computing and the 2019 ACM International Symposium on Wearable Computers (UbiComp/ISWC).

[19] Young A.D., Ling M.J., Arvind D.K. IMUSim: A Simulation Environment for Inertial Sensing Algorithm Design and Evaluation. Proceedings of the 2011 10th International Conference on Information Processing in Sensor Networks (IPSN).

[20] Stanley M. Open Source IMU Simulator, Trajectory & Sensor Simulation Toolkit. Freescale Semiconductor & MEMS Industry Group Accelerated Innovation Community. https://github.com/memsindustrygroup/TSim.

[21] Kwon H., Tong C., Gao Y., Abowd G.D., Lane N.D., Plötz T., Haresamudram H. (2020). IMUTube: Automatic Extraction of Virtual on-Body Accelerometry from Video for Human Activity Recognition. Proc. ACM Interact. Mob. Wearable Ubiquitous Technol..

[22] Gadaleta M., Rossi M. (2018). IDNet: Smartphone-Based Gait Recognition with Convolutional Neural Networks. Pattern Recognit..

[23] Zou Q., Wang Y., Wang Q., Zhao Y., Li Q. (2018). Deep Learning-Based Gait Recognition Using Smartphones in the Wild. IEEE Trans. Inf. Forensics Secur..

[24] Huang H., Zhou P., Li Y., Sun F. (2021). A Lightweight Attention-Based CNN Model for Efficient Gait Recognition with Wearable IMU Sensors. Sensors.

[25] Mahmood Hussein M., Hussein Mutlag A., Shareef H., Pu J., Zhang L., Zhang J., Teoh K., Ismail R., Naziri S., Hussin R. (2021). Face Recognition and Identification Using Deep Learning Approach. J. Phys. Conf. Ser..

[26] Matovski D.S., Nixon M.S., Mahmoodi S., Carter J.N. (2012). The Effect of Time on Gait Recognition Performance. IEEE Trans. Inf. Forensics Secur..

[27] Topham L.K., Khan W., Al-Jumeily D., Hussain A. (2023). Human Body Pose Estimation for Gait Identification: A Comprehensive Survey of Datasets and Models. arXiv.

